# Deep Learning-Based Full-Process Automatic CPAK Classification System and Its Application in the Analysis of Alignment Outcomes Before and After Knee Arthroplasty

**DOI:** 10.3390/diagnostics16091389

**Published:** 2026-05-03

**Authors:** Kun Wu, Xiao Geng, Xinguang Wang, Jiazheng Chen, Hua Tian

**Affiliations:** 1 Orthopaedic Department, Peking University Third Hospital, No. 49 North Garden Road, Beijing 100191, China; wukunputh@163.com (K.W.); gengxiao@bjmu.edu.cn (X.G.); wangxinguang@bjmu.edu.cn (X.W.); 2 Department of Orthopaedic, The First Affiliated Hospital, Zhejiang University School of Medicine, No. 79 Qingchun Road, Hangzhou 310003, China; chenjiazheng@bjmu.edu.cn

**Keywords:** total knee arthroplasty, CPAK classification, deep learning, alignment transition, individualized alignment, HRNet-W32

## Abstract

**Background/Objectives**: Coronal Plane Alignment of the Knee (CPAK) classification enables individualized alignment assessment in total knee arthroplasty (TKA), yet manual evaluation is time-consuming and lacks preoperative-to-postoperative transition analysis. **Methods**: This retrospective, single-center study aimed to develop and validate a fully automated deep learning-based CPAK classification system using internal validation on a held-out test set (*n* = 92) and to investigate individual-level transition patterns and their association with short-term clinical outcomes using paired radiographic data from a large Chinese cohort. A total of 919 KOA patients undergoing TKA were analyzed. A keypoint detection model (HRNet-W32) was developed to automatically calculate the medial proximal tibial angle, lateral distal femoral angle, arithmetic hip-knee-ankle angle, and joint line obliquity, from which CPAK types were derived. **Results**: On the validation set (92 cases), the model achieved a Mean Radial Error of 1.22 ± 0.43 mm for keypoint detection; mean absolute errors for MPTA and LDFA were ≤0.74°, while for aHKA and JLO they were 0.91° and 1.12°, respectively, with intraclass correlation coefficients ≥0.96 compared to manual annotations. Automatic CPAK classification accuracy was 80.98% (kappa = 0.767). Transition matrix analysis showed that only 9.36% of all patients maintained their original type postoperatively, with most shifting to types IV, V, or VII. After inverse probability weighting, no significant differences in clinical outcomes were observed among transition groups (all adjusted *p* > 0.05). **Conclusions**: These results demonstrate that the proposed automated system enables efficient CPAK assessment, revealing substantial postoperative alignment transitions that were not associated with differential short-term outcomes, thereby supporting AI-assisted individualized alignment planning in TKA.

## 1. Introduction

Knee osteoarthritis (KOA) is a leading cause of functional impairment in the middle-aged and elderly population. Total knee arthroplasty (TKA), as an effective treatment for end-stage KOA, can significantly relieve pain and improve function [1,2]. Postoperative outcomes of TKA are closely related to the reconstruction of lower limb alignment, which directly affects prosthesis stress distribution, joint stability, and long-term patient prognosis [3,4]. Conventional TKA aims to restore mechanical alignment (MA) as its core objective. Although its 10-year implant survival rate can exceed 90%, approximately 20% of patients remain dissatisfied with postoperative outcomes [5,6,7,8]. This is primarily because MA does not fully account for individual anatomical variations, potentially leading to soft tissue imbalance or abnormal joint line obliquity [9,10]. Consequently, alternative individualized strategies have been proposed, including anatomic alignment (AA), kinematic alignment (KA), restricted kinematic alignment (rKA), and functional alignment (FA) [11,12,13,14]. However, AA has limited applicability for complex deformities; KA may result in alignment deviations >3° with insufficient long-term evidence; the safe range for rKA remains undefined; and FA relies heavily on surgeon experience and navigation equipment, limiting its widespread adoption [15,16].

The core of individualized alignment strategies lies in identifying distinct anatomical phenotypes among patients [17,18]. In 2021, MacDessi et al. introduced the Coronal Plane Alignment of the Knee (CPAK) classification system [19]. This system utilizes the medial proximal tibial angle (MPTA) and lateral distal femoral angle (LDFA) to calculate the arithmetic hip-knee-ankle angle (aHKA) and joint line orientation (JLO), classifying the knee into nine phenotypes and providing a systematic framework for personalized surgical planning [19,20,21]. A deeper understanding of the anatomical characteristics of CPAK types and their association with postoperative outcomes is crucial for optimizing surgical strategies [22,23].

However, the clinical implementation of this classification faces bottlenecks. Currently, CPAK classification relies on manual measurements, which are time-consuming and subjective, typically requiring approximately 3 min per case [24] and showing interobserver ICC values ranging from 0.78 to 0.99 [14], thus limiting its widespread clinical application [25]. Furthermore, as a two-dimensional radiographic classification, CPAK inherently does not capture sagittal plane alignment, rotational deformities, or soft tissue balance, all of which are recognized as important contributors to postoperative knee function. In recent years, deep learning has made significant strides in medical image analysis, particularly excelling in automated skeletal keypoint detection and angle measurement [26,27]. Studies have shown that fully automated alignment measurement systems based on convolutional neural networks achieve excellent agreement with manual annotations, with intraclass correlation coefficients (ICC) > 0.90, laying the technical groundwork for automating CPAK classification [28]. Recent advances have further expanded the application of deep learning in orthopedic image analysis. For instance, the ResNet-based HKA-Net has demonstrated efficient HKA angle measurement without explicit landmark annotation [29]. While developed for chest radiographs, the spatial dependency modeling in architectures such as SPX-GNN highlights transferable strategies for long-bone analysis [30]. The growing role of graph neural networks in medical imaging has also been systematically reviewed [31]. These recent developments underscore the timeliness and relevance of our work in developing an automated CPAK classification system for large-scale clinical application.

Current research on the Chinese population has reported the distribution characteristics of CPAK types [32]; however, the individual-level transition pathways from preoperative to postoperative CPAK types remain unclear [9,10,11]. Key bottlenecks include the following: the acquisition of CPAK types still relies on manual effort, hindering large-scale application, and the individual-level transition patterns from preoperative to postoperative CPAK types have not been clarified. To address these gaps, this study aims to develop a fully automated deep learning-based CPAK classification system and, using paired pre- and postoperative data from a large cohort of Chinese TKA patients, systematically analyze the individual-level transition patterns of CPAK types and their association with clinical outcomes. The specific objectives are: 1. To develop and validate a fully automated CPAK classification model; 2. To construct a transition matrix to elucidate transition patterns; 3. To analyze the impact of different transition patterns on clinical outcomes.

## 2. Materials and Methods

This study was approved by the Medical Science Research Ethics Committee of Peking University Third Hospital (IRB00006761-M2023429) and was registered in ClinicalTrials.gov (NCT06010979).

### 2.1. Study Design and Population

A total of 1000 patients with knee osteoarthritis who underwent TKA at Peking University Third Hospital between January and December 2023 were initially identified. All patients had pre- and postoperative full-length weight-bearing anteroposterior lower limb radiographs. Patients were randomly divided into a model development set (*n* = 827, 1654 radiographs) and a model validation set (*n* = 92, 184 radiographs) at the patient level. Inclusion criteria were as follows: Kellgren-Lawrence grade ≥ 3; underwent TKA; and had standard pre- and postoperative full-length weight-bearing lower limb radiographs. Exclusion criteria were as follows: images with flexion or rotational deformity (rotation > 10°); severe bone defects on the femoral or tibial side; and poor image quality. A total of 919 patients were ultimately included for analysis. Patient demographic information, including age, sex, height, weight, body mass index (BMI), surgical technique, and preoperative Kellgren-Lawrence grade of the affected side, was collected from the medical records.

### 2.2. Clinical Outcome Assessment

Postoperative evaluation was conducted through outpatient follow-up or telephone interviews, with a follow-up period of 12–24 months. Assessment tools included the Knee Society Score (KSS), the Western Ontario and McMaster Universities Osteoarthritis Index (WOMAC), the Forgotten Joint Score (FJS), and a 5-level Likert satisfaction scale (for statistical analysis, “satisfied” and “very satisfied” were combined into the “satisfied” group, while the remaining responses were combined into the “non-satisfied” group). All clinical outcome data were collected by independent researchers who were not involved in the surgical procedures or image annotation.

### 2.3. Image Acquisition, Manual Annotation, and Gold Standard

Standard full-length weight-bearing lower limb radiographs were acquired. Key anatomical landmarks were defined according to MacDessi et al. [19], including the femoral head center, femoral intercondylar notch center, distal medial and lateral femoral condyle points, tibial intercondylar eminence center, medial and lateral tibial plateau lowest points, and talar dome center (ankle joint center).

**Figure 1:**
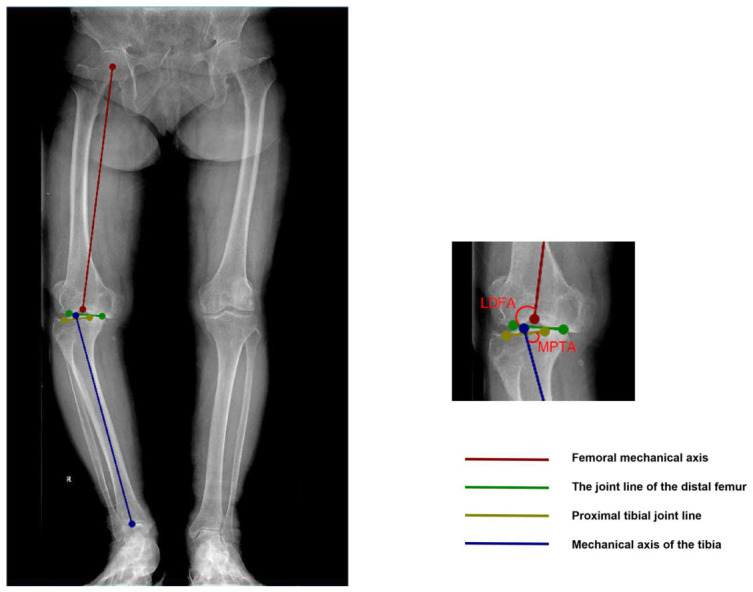
Labelme annotation diagram and schematic diagrams of LDFA and MPTA.

One orthopedic resident manually annotated these keypoints using Labelme software(v5.4.1) (Figure 1), and two senior chief physicians reviewed and corrected the annotations. For the gold standard, three senior chief physicians, blinded to the AI results, independently performed manual measurements (MPTA, LDFA, aHKA, JLO) and CPAK classification using professional imaging software. The final classification was based on the consensus of at least two experts. Kendall’s coefficient of concordance (W) was used to assess inter-observer consistency, with W > 0.8 indicating extremely high agreement. The mean values of the three experts were used as the reference for evaluating the accuracy of the AI measurements. We expected Kendall’s W > 0.8 to indicate high inter-observer consistency. The reliability of manual landmark annotation on lower limb radiographs has been well established in previous studies [14,19].

### 2.4. CPAK Type Calculation

**Figure 2:**
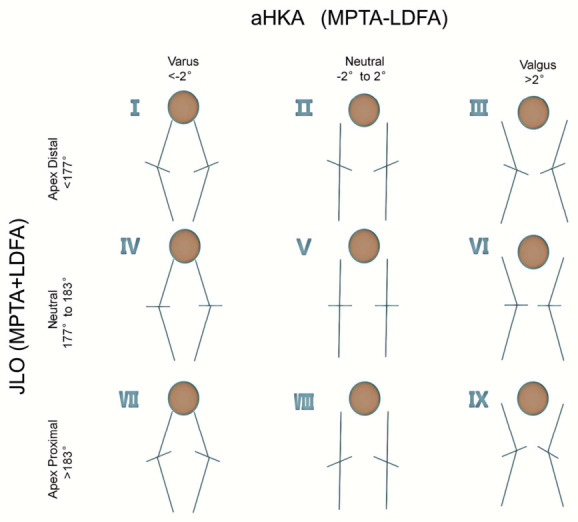
The nine phenotypes of knee coronal plane alignment (CPAK classification).

The formulas for CPAK type calculation were aHKA = MPTA − LDFA and JLO = MPTA + LDFA. Classification thresholds were as follows: aHKA < −2° was classified as varus, −2° to +2° as neutral, and >+2° as valgus; JLO < 177° was classified as apex distal, 177–183° as neutral, and >183° as apex proximal. Preoperative and postoperative CPAK types (I–IX) were determined accordingly (Figure 2).

### 2.5. Deep Learning-Based Automatic CPAK Classification System

#### 2.5.1. Overall Workflow and Image Preprocessing

The deep learning-based automatic CPAK classification system developed in this study consisted of three main stages: image preprocessing, keypoint detection, and angle calculation with CPAK type output. During preprocessing, original full-length weight-bearing lower limb radiographs underwent side identification; left knee images were horizontally flipped to standardize them to the right knee orientation, eliminating the impact of anatomical differences between sides. Flipping parameters were recorded for subsequent coordinate mapping, and all images were uniformly resized to a fixed dimension for network input. During model training, data augmentation strategies including random flipping, rotation, and brightness adjustment were further applied to enhance model generalization.

#### 2.5.2. Keypoint Detection

**Figure 3:**
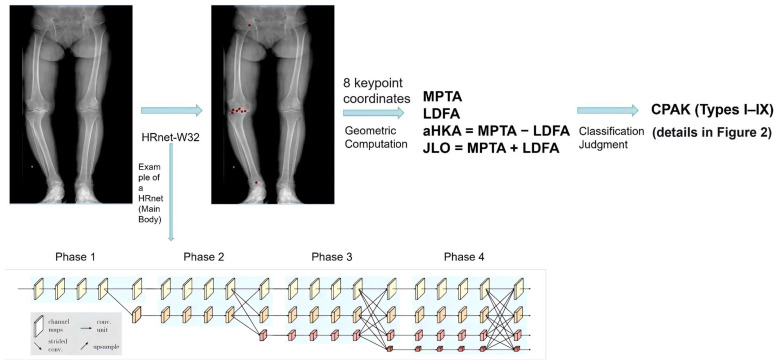
Schematic workflow of the proposed automated CPAK classification system. Input full-length weight-bearing radiographs are processed by the HRNet-W32 network to detect eight anatomical keypoints. The coordinates of these keypoints are then used to compute MPTA, LDFA, aHKA and JLO according to the geometric formulas shown in the figure. Finally, the CPAK type (I–IX) is determined based on the aHKA and JLO thresholds (detailed in Figure 2). The diagram illustrates the complete pipeline from image to classification.

This study employed the HRNet-W32 network for keypoint detection, a deep neural network architecture based on the High-Resolution Network (HRNet) [33] that achieves an excellent balance between computational efficiency and accuracy. The network consists of four parallel subnetworks across four stages: each stage introduces a subsequent subnetwork, the number of channels in parallel branches doubles while resolution decreases by 50%, and fusion modules connect the stages to exchange and fuse feature information from different resolutions, thereby maintaining high-resolution representations while capturing rich semantic features (Figure 3). For this specific task, a convolutional layer with the corresponding number of channels was added as the output layer on top of HRNet-W32 to predict eight keypoint heatmaps (femoral head center, femoral intercondylar notch center, distal medial/lateral femoral condyle points, tibial intercondylar eminence center, medial/lateral tibial plateau lowest points, and talar dome center). Transfer learning was applied using pre-trained weights from an image classification task; after Gaussian transformation, the annotated keypoint coordinates were converted into heatmap labels for training. The trained model outputs heatmaps, which are then converted into keypoint coordinate values, thereby enabling automatic detection of the eight anatomical keypoints. The output layer was modified to predict eight keypoint heatmaps using a convolutional layer with eight filters. The model was trained using the Adam optimizer with an initial learning rate of 0.001, batch size of 16, and 210 epochs. The loss function was mean squared error (MSE) between predicted heatmaps and Gaussian-labeled ground-truth heatmaps (σ = 2 pixels). All experiments were conducted on an NVIDIA GeForce RTX 3060 laptop GPU (NVIDIA, Santa Clara, CA, USA). Specifically, random rotation was applied within ±10°, and brightness was randomly scaled by a factor of 0.8–1.2.

#### 2.5.3. Angle Calculation, CPAK Classification, Dataset Partitioning, and Evaluation Metrics

Based on the detected keypoint coordinates, MPTA, LDFA, aHKA, and JLO were geometrically calculated (see Section 2.4 for formulas), and the CPAK type (I–IX) was automatically determined based on the aHKA and JLO thresholds. All samples were divided at the patient level into a model development set (*n* = 827, 1654 radiographs) and a model validation set (*n* = 92, 184 radiographs). The development set was used for training and internal validation (using cross-validation), while the validation set was used to evaluate keypoint detection accuracy (Mean Radial Error, MRE), angle measurement accuracy (mean absolute error, MAE, and standard deviation), agreement with manual annotations (intraclass correlation coefficients, ICC, and Bland–Altman plots), and CPAK classification performance (overall accuracy, recall per type, and Cohen’s kappa coefficient). The validation set was also included in the subsequent clinical analyses (CPAK distribution, transition matrix, outcome association) to maintain sample representativeness; because it was not used for any parameter updates or model selection during the development process, this inclusion avoided information leakage.

To further confirm the robustness of our clinical findings, a sensitivity analysis was performed using only the development set (*n* = 827) for all clinical analyses (CPAK distribution, transition matrix, transition pattern grouping, and outcome comparisons). The results were consistent with the main analysis and are provided in the Supplementary Materials (Tables S1–S4).

### 2.6. Preoperative-to-Postoperative CPAK Type Transition Analysis

After model performance was validated, all 1838 radiographs from the 919 patients were input into the trained deep learning model, which automatically output angle measurements and CPAK types for each image. This part of the analysis was entirely based on model predictions without manual verification. To describe individual-level transitions, a 9 × 9 contingency table was constructed with rows representing preoperative types and columns representing postoperative types, and frequencies and row percentages were calculated. Based on the transition characteristics between preoperative and postoperative CPAK types, patients were divided into four groups: the Stable group (preoperative and postoperative types identical), the Alignment-changed group (only aHKA classification changed, JLO classification unchanged), the Joint line-changed group (only JLO classification changed, aHKA classification unchanged), and the Mixed-changed group (both aHKA and JLO classifications changed). This grouping was designed to isolate the independent contributions of alignment (aHKA) and joint line obliquity (JLO) changes to clinical outcomes, leveraging the two-dimensional nature of the CPAK classification. As no standardized guidelines currently exist for categorizing CPAK transition patterns, this grouping serves as an exploratory framework for hypothesis generation.

### 2.7. Association Analysis Between Transition Patterns and Clinical Outcomes

Due to baseline differences among the four groups, we used inverse probability of treatment weighting (IPTW) to balance covariates. A multinomial logistic regression model was fitted with transition group (Stable, Alignment-changed, Joint line-changed, Mixed-changed) as the dependent variable and the following covariates as independent variables: age, sex, BMI, preoperative K-L grade, and surgical technique. For each patient, the propensity score was defined as the predicted probability of belonging to his/her actual group. Inverse probability weights were calculated as w = 1/propensity score, and weights were truncated at the 1st and 99th percentiles to avoid extreme values. Covariate balance after weighting was assessed using standardized mean differences (SMD), with SMD < 0.1 indicating good balance. Weighted group comparisons for clinical outcomes were performed using weighted Kruskal–Wallis tests (for KSS, WOMAC, FJS) and weighted chi-square tests (for satisfaction).

### 2.8. Statistical Analysis

SPSS 27.0 was used for data analysis and Origin 2024 for graph generation. Kendall’s coefficient of concordance was used to evaluate consistency among expert measurements, and intraclass correlation coefficients (ICC) were used to assess agreement between model measurements and manual annotations. Continuous variables following a normal distribution were expressed as mean ± standard deviation ($\bar{x}$ ± s), while non-normally distributed variables were expressed as median (interquartile range); categorical variables were expressed as frequencies (percentages). Group comparisons were performed using an independent samples *t*-test, Mann–Whitney U test, or chi-square (χ^2^) test, and the χ^2^ test was also used for the dichotomized satisfaction variable. Multiple group comparisons were performed using one-way ANOVA or the Kruskal–Wallis test. A two-sided *p*-value < 0.05 was considered statistically significant.

## 3. Results

### 3.1. Patient Baseline Characteristics

A total of 919 patients undergoing TKA for knee osteoarthritis were included in this study. Among them, there were 215 males (23.4%) and 704 females (76.6%). The mean age was 68.03 ± 6.23 years (range: 34–84 years), and the mean BMI was 26.85 ± 3.50 kg/m^2^. Regarding K-L grades, 310 patients (33.73%) were grade 3, and 609 patients (66.27%) were grade 4. All patients completed clinical follow-up of 12–24 months postoperatively. Robotic-assisted surgery was performed in 217 cases (23.6%) and conventional surgery in 702 cases (76.4%).

### 3.2. Performance Validation of the Deep Learning Model

#### 3.2.1. Keypoint Detection Accuracy

On the model validation set (92 patients, 184 radiographs), the HRNet-W32 model achieved a Mean Radial Error (MRE) of 1.22 ± 0.43 mm for the eight anatomical keypoints. The detection accuracy for each keypoint is shown in Table 1. The femoral head center and the ankle joint center had the smallest detection errors, while the errors for the medial and lateral tibial plateau points were relatively larger.

#### 3.2.2. Angle Measurement Accuracy

A comparison between the model’s automatically measured angles and the gold standard annotations is shown in Table 2. The mean absolute error (MAE) for MPTA was 0.74 ± 0.42° (8.70% of errors ≥ 1.5°), for LDFA was 0.72 ± 0.51° (15.22% of errors ≥ 1.5°), for aHKA was 0.91 ± 0.70° (19.57% of errors ≥ 1.5°), and for JLO was 1.12 ± 0.66° (22.28% of errors ≥ 1.5°).

#### 3.2.3. Agreement with Manual Annotations

The three orthopedic surgeons demonstrated extremely high consistency in measuring MPTA, LDFA, aHKA, and JLO angle (all W > 0.95, all *p* < 0.05). Their mean values were as follows: MPTA: 87.19 ± 3.90°, LDFA: 90.01 ± 3.22°, aHKA: −2.82 ± 4.78°, JLO: 177.20 ± 5.33°. The automatic model measurements were as follows: MPTA: 86.72 ± 3.93°, LDFA: 89.67 ± 3.25°, aHKA: −2.96 ± 4.88°, JLO: 176.39 ± 5.30°.

**Figure 4:**
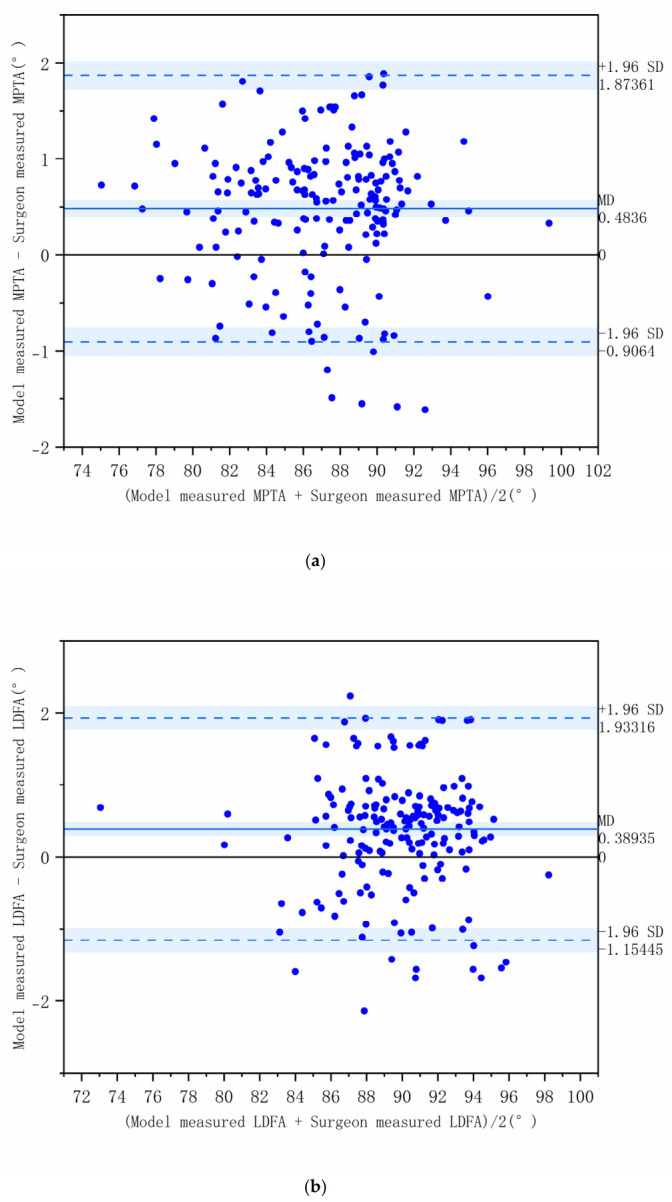

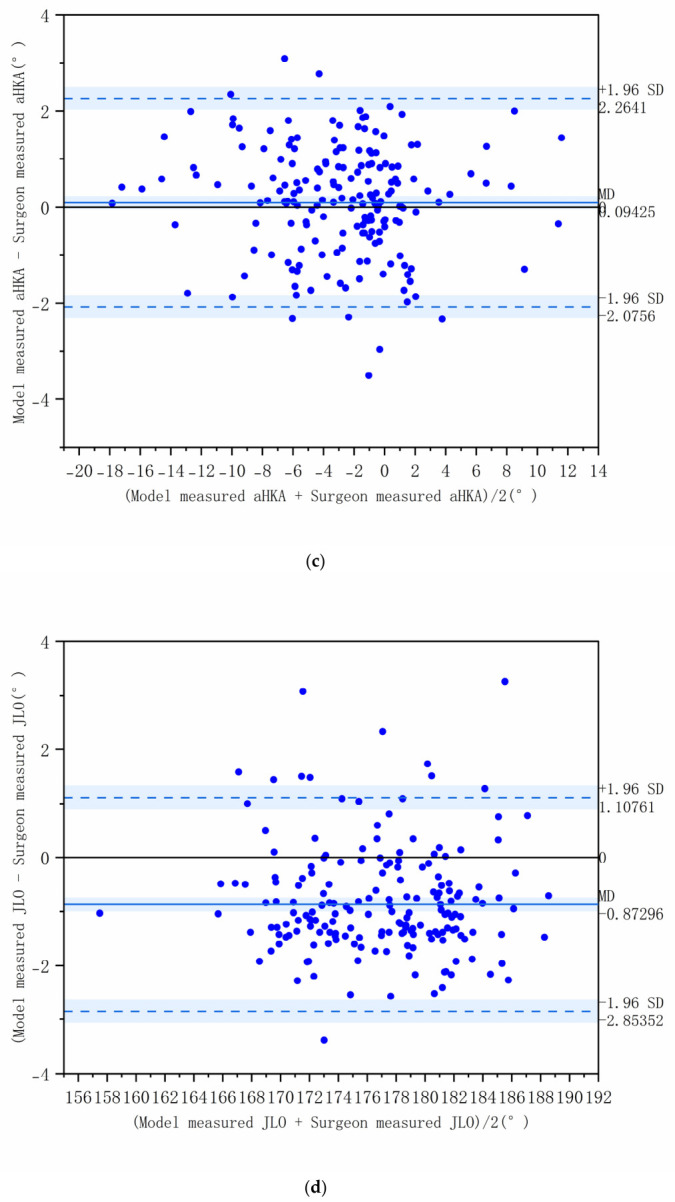
(**a**–**d**) Bland–Altman plots for assessing agreement of model-predicted angles on the validation set. (**a**) Medial proximal tibial angle (MPTA); (**b**) Lateral distal femoral angle (LDFA); (**c**) Arithmetic hip-knee-ankle angle (aHKA); (**d**) Joint line obliquity (JLO). The solid line represents the mean difference, and the dashed lines indicate the 95% limits of agreement.

ICC analysis showed excellent agreement between model measurements and manual annotations: MPTA ICC = 0.977 (95% CI: 0.930–0.990); LDFA ICC = 0.963 (95% CI: 0.940–0.980); aHKA ICC = 0.972 (95% CI: 0.960–0.980); JLO ICC = 0.970 (95% CI: 0.880–0.990) (all *p* < 0.001). As shown in the Bland–Altman plots (Figure 4a–d), the measurement differences for each angle fell within the 95% limits of agreement, indicating no systematic bias.

#### 3.2.4. Automatic CPAK Type Classification Evaluation

Using the manually annotated CPAK type as the gold standard, the overall accuracy of the model’s automatic classification was 80.98%. Cohen’s kappa coefficient was 0.767. The recall rates for each type are shown in Table 3 (type VIII had a low recall due to the small sample size), indicating good overall performance of the automatic CPAK classification and high agreement with expert judgment.

### 3.3. Preoperative and Postoperative CPAK Type Distribution

The trained deep learning model was applied to automatically measure the images of all 919 patients, yielding the corresponding angle parameters and CPAK types. The distribution of preoperative and postoperative CPAK types among the 919 patients is shown in Table 4. Preoperatively, the most common types were Type I (535 cases, 58.22%), followed by Type II (139 cases, 15.13%), Type IV (97 cases, 10.55%), and Type III (75 cases, 8.16%). Types V–IX were relatively rare, collectively accounting for 7.94%. Postoperatively, the most common types were Type V (283 cases, 30.79%), Type IV (263 cases, 28.62%), and Type VII (137 cases, 14.91%), collectively accounting for 74.32%.

### 3.4. Individual-Level Preoperative-to-Postoperative CPAK Type Transition Matrix

**Figure 5:**
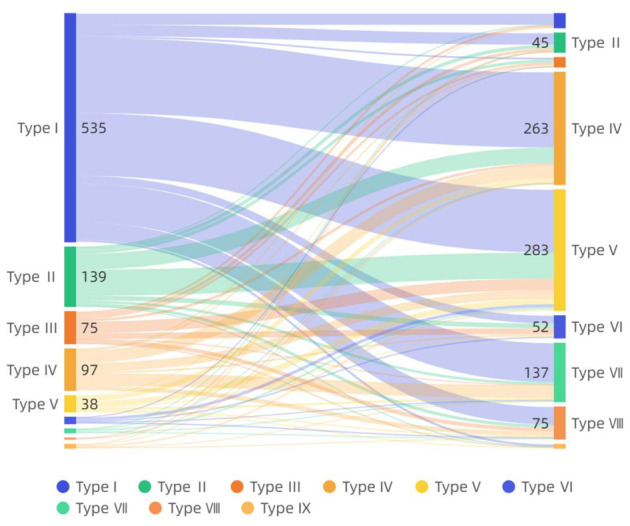
Sankey diagram of preoperative-to-postoperative CPAK type transitions.

An individual-level transition matrix from preoperative to postoperative CPAK types was constructed (Table 5, row percentages). The Stuart-Maxwell test revealed a significant difference in the overall distribution between preoperative and postoperative CPAK types (χ^2^ = 647.2, df = 8, *p* < 0.001). Based on the transition patterns, patients were divided into four groups: the Stable group (preoperative and postoperative types identical) comprised 86 cases (9.36%); the Alignment-changed group (only aHKA classification changed) comprised 113 cases (12.30%); the Joint line-changed group (only JLO classification changed) comprised 381 cases (41.45%); and the Mixed-changed group (both aHKA and JLO classifications changed) comprised 339 cases (36.89%). Figure 5 presents a Sankey diagram delineating the predominant migration streams from preoperative phenotypes to their postoperative CPAK counterparts. Of note, the 92 patients from the validation set were included in this transition analysis to maximize sample representativeness; however, sensitivity analyses using only the development set (*n* = 827) yielded similar patterns (Supplementary Tables S1–S4).

Sensitivity analysis using only the development set yielded similar transition patterns (see Supplementary Table S2).

### 3.5. Association Between Transition Patterns and Clinical Outcomes

#### 3.5.1. Baseline Balance After Inverse Probability Weighting

After applying IPTW, all covariates were well balanced across the four groups (Table 6). Weighted comparisons showed no significant differences in any of the clinical outcomes (Table 7).

#### 3.5.2. Comparison of Clinical Outcomes Between Groups

The comparison of clinical outcomes among the four groups after IPTW is presented in Table 7: Overall satisfaction rates were above 90% in all four groups, with no significant difference (*p* > 0.05); similarly, no significant differences were observed among the four groups in KSS, WOMAC score, or FJS (all *p* > 0.05).

A post hoc power analysis was performed for the comparison of FJSs between the Stable and Alignment-changed groups using G*Power (version 3.1). With an observed effect size (Cohen’s d) of 0.35, α = 0.05, and sample sizes of 86 per group, the achieved power was 0.62, which is below the conventional threshold of 0.80. This indicates that the study may have been underpowered to detect small differences, and the lack of statistical significance should be interpreted cautiously. Similar power values were obtained for comparisons with the other groups. Larger sample sizes are warranted in future studies.

## 4. Discussion

In this study, we developed and validated a fully automated CPAK classification system based on HRNet-W32 and applied it to analyze individual-level preoperative-to-postoperative transition patterns. We systematically investigated the distribution characteristics of CPAK types, their transition patterns, and their association with clinical outcomes. The main findings and their clinical implications are discussed below.

### 4.1. Performance and Value of the Deep Learning-Based Automatic CPAK Classification System

Using HRNet-W32 as the backbone network for keypoint detection, our model achieved a Mean Radial Error (MRE) of 1.22 ± 0.43 mm on the validation set. The femoral head center and talar center exhibited the highest detection accuracy, while errors for the medial and lateral tibial plateau points were relatively larger, consistent with previous reports indicating higher difficulty in detecting distal keypoints [34]. Benchmarking against prior work, our MRE of 1.22 mm compares favorably with the 1.57 mm reported by Moon et al. using a U-Net variant, while our ICCs (≥0.96) align with the high agreement metrics (0.95–0.98) documented by Lee et al. These metrics confirm the system’s reliability for CPAK classification [14,34]. In terms of angle measurement, the MAE for MPTA and LDFA were 0.74° and 0.72°, respectively, while those for aHKA and JLO were 0.91° and 1.12°, respectively. The ICCs with manual annotations were all above 0.96, and Bland–Altman plots showed no systematic bias, indicating excellent clinical consistency.

At the CPAK type level, the overall accuracy of automatic classification was 80.98%, with a kappa coefficient of 0.767, suggesting high agreement with expert judgment. Notably, the recall rate for some rare types (e.g., type VIII) was lower, likely due to their sparse distribution in the training set. This suggests that future improvements could focus on data augmentation or class weighting strategies to enhance model generalizability for rare phenotypes. Overall, the measurement accuracy and classification consistency of this system reached an acceptable level, reducing analysis time per full-length radiograph from 3 to 5 min manually to seconds, providing an efficient and objective tool for large-scale clinical studies and personalized surgical planning.

However, these rare types are extremely uncommon in clinical practice: in our preoperative cohort, Type VIII accounted for only 0.22% (2/919) and Type VI for 1.63% (15/919). Therefore, even with suboptimal performance on these rare phenotypes, the clinical impact on overall patient management is minimal. When evaluating the model on the remaining common types (I–V, VII, IX), the classification accuracy reached 84.8%. We have added precision and F1-scores for all types in Table 3 to provide a more complete assessment. We agree that further improvement is desirable and plan to address class imbalance in future work using data augmentation or synthetic oversampling techniques (e.g., SMOTE). In its current state, the system already offers substantial benefit for the vast majority of cases and can be safely used as an assistive tool in clinical practice.

### 4.2. Distribution Characteristics of Preoperative and Postoperative CPAK Types in the Chinese TKA Population

The automatic classification results revealed that Type I was the predominant preoperative CPAK type in Chinese KOA patients (58.22%), followed by Types II and IV, with Type V accounting for only 4.13%. This distribution significantly differs from the predominantly Type II distribution reported by MacDessi et al. in an Australian population [19], highlighting the heterogeneity of knee anatomical phenotypes across different ethnic groups. Compared with reports from Japanese and Indian populations, our results align more closely with the actual distribution in mainland Chinese populations, further confirming the significant racial and regional specificity of the CPAK classification [9,35,36]. These findings are consistent with our team’s previous research [32].

The postoperative CPAK type distribution changed significantly (*p* < 0.001), with Types V, IV, and VII predominating, collectively accounting for 74.32%. This reflects the general postoperative “correction” of alignment toward a neutral alignment or mild varus with altered joint line obliquity, consistent with current surgical strategies guided by mechanical or functional alignment principles.

### 4.3. Individual-Level Preoperative-to-Postoperative CPAK Type Transition Patterns

Based on individual paired data, we constructed a preoperative-to-postoperative CPAK type transition matrix, revealing detailed pathways of alignment transition. The results showed that patients with the most common preoperative Type I primarily transitioned to Types IV, V, and VII postoperatively, indicating that most patients with varus knees were corrected to a neutral or mild varus alignment after TKA, though some retained a varus phenotype. Preoperative Type II patients mainly transitioned to Types V and IV, while preoperative Type III patients predominantly transitioned to Types V and VI. According to the transition pattern grouping, only 9.36% of patients maintained their original type postoperatively, while the Joint line-changed and Mixed-changed groups together accounted for 78.34%. This demonstrates that TKA significantly impacts the natural coronal alignment characteristics of patients. This finding provides a comprehensive view of postoperative alignment transition and offers quantitative evidence for developing individualized alignment strategies.

### 4.4. Association Between Transition Patterns and Clinical Outcomes

This study found no statistically significant differences in postoperative clinical outcomes among different transition patterns. After inverse probability weighting, the Stable, Alignment-changed, Joint line-changed, and Mixed-changed groups showed no significant differences in KSS, WOMAC, FJSs, or satisfaction (*p* > 0.05). This result partially aligns with the study by Sappey-Marinier et al., which also found that postoperative changes in joint line obliquity did not significantly affect clinical scores [17]. Notably, although the FJS score differences did not reach statistical significance, the mean values in the Alignment-changed and Mixed-changed groups were higher than those in the Stable group, suggesting a potential clinically meaningful trend that warrants further investigation with larger sample sizes or longer follow-up periods. The lack of a significant impact of transition patterns on clinical outcomes might be related to the relatively short follow-up period (12–24 months) and the high individual variability in patient-reported outcomes. It may also indicate that in the short term, patient satisfaction is more influenced by overall surgical outcomes such as pain relief and functional recovery, rather than by the specific pattern of alignment or joint line change.

### 4.5. Innovations and Clinical Significance

The innovations of this study are primarily reflected in the following aspects: it combined deep learning-based automatic CPAK classification with individual-level paired pre- and postoperative analyses, achieving fully automated assessment of alignment transition; it constructed a CPAK type transition matrix based on a large sample (919 cases) of paired pre- and postoperative data from the Chinese population, revealing individual-level transition patterns; and it systematically analyzed the association between different transition patterns and clinical outcomes, providing real-world data support for selecting individualized alignment strategies. Seamless integration into existing Picture Archiving and Communication Systems (PACS) enables real-time, point-of-care phenotyping. This facilitates rapid identification of patients whose native alignment deviates from conventional mechanical targets, thereby informing individualized strategy selection (e.g., kinematic or restricted kinematic alignment) preoperatively. Furthermore, the transition matrix provides a quantitative reference for expected postoperative alignment shifts, which can aid in managing patient expectations and evaluating surgical consistency.

### 4.6. Limitations

This study has several limitations. It is a single-center retrospective design; all data came from one medical center, which may introduce selection bias, and the generalizability of the conclusions needs to be verified. The follow-up period was relatively short (12–24 months); the association between long-term implant survival and alignment transition remains to be investigated. Patient-reported outcomes are subjective; FJSs, KSSs, etc., may be influenced by subjective patient factors and postoperative rehabilitation differences, potentially introducing information bias. Additionally, the study cohort comprised 76.6% female patients, which is consistent with the well-documented higher prevalence of knee osteoarthritis in women. However, this sex imbalance may limit the generalizability of our findings to male patients and could affect the statistical power for sex-stratified analyses. The generalizability of the deep learning model may be limited; the model was trained on images from a single center, and its adaptability to different equipment and imaging conditions requires external validation with multi-center data.

Moreover, the validation set was relatively small (*n* = 92), and no external validation was performed. Therefore, the generalizability of the model to other centers or imaging protocols remains to be established. Furthermore, the deployment of deep learning models requires dedicated computational resources (e.g., GPU), which may not be universally available in all clinical settings. In addition, the model was developed and validated exclusively on standardized full-length weight-bearing radiographs; its performance on other image types (e.g., supine or non-weight-bearing views) remains unknown and warrants further investigation. Future studies with larger multi-center datasets are needed to confirm the robustness of our findings. In this study, the model validation set was included in the clinical analysis to maintain the completeness and representativeness of the clinical sample. Although this set was not used for model training or validation, and theoretically does not introduce overfitting bias, strictly adhering to the principle of an independent validation set would require its exclusion from subsequent analyses. This set was retained primarily to maximize the clinical sample size, and as the randomly divided validation set and clinical analysis set showed no significant baseline differences (SMD < 0.1), the impact on the main conclusions is likely limited. Although we used inverse probability weighting to balance covariates, residual confounding due to unmeasured factors (e.g., activity level, socioeconomic status) cannot be completely ruled out. Future prospective studies with larger sample sizes and more comprehensive covariate collection are warranted to confirm our findings. The CPAK classification itself has limitations: it focuses only on the coronal plane and does not incorporate factors such as sagittal or rotational alignment or soft tissue balance, which also significantly impact postoperative function. Future research directions include conducting multicenter prospective studies with extended follow-up and incorporating more imaging and functional indicators; exploring the combined application value of CPAK classification with other alignment strategies (e.g., kinematic alignment and functional alignment); and further optimizing deep learning models to improve the identification of rare phenotypes.

## 5. Conclusions

This study, utilizing a deep learning-based automatic CPAK classification system, revealed that the predominant preoperative CPAK type in Chinese TKA patients is Type I, while postoperative types IV, V, and VII are most common. An individual-level transition matrix was constructed, demonstrating that most patients undergo significant changes in alignment and joint line post-TKA. However, no statistically significant differences in short-term clinical outcomes were observed among different transition patterns. These findings provide data-driven support for individualized TKA alignment strategies and offer a feasible tool for artificial intelligence-assisted preoperative planning and postoperative evaluation.

## Figures and Tables

**Table 1:** Keypoint detection accuracy (MRE).

Keypoint	MRE (mm)
Femoral head center	0.87 ± 0.31
Femoral intercondylar notch center	1.02 ± 0.38
Distal medial femoral condyle point	1.34 ± 0.45
Distal lateral femoral condyle point	1.29 ± 0.42
Tibial intercondylar eminence center	1.41 ± 0.51
Medial tibial plateau lowest point	1.68 ± 0.62
Lateral tibial plateau lowest point	1.72 ± 0.65
Talar dome center	0.53 ± 0.22

**Table 2:** Angle measurement error analysis.

Angle	MAE (°)	Proportion of Errors ≥ 1.5° (%)
MPTA	0.74 ± 0.42	8.70
LDFA	0.72 ± 0.51	15.22
aHKA	0.91 ± 0.70	19.57
JLO	1.12 ± 0.66	22.28

**Table 3:** Performance metrics for each CPAK type on the model validation set (184 radiographs, 92 patients).

CPAK Type	True Number	Correctly Predicted (TP)	Recall (%)	Precision (%)	F1-Score (%)
I	51	49	96.08	94.23	95.15
II	27	23	85.19	82.14	83.64
III	8	6	75.00	66.67	70.59
IV	32	26	81.25	78.79	80.00
V	38	29	76.32	74.36	75.32
VI	6	3	50.00	37.50	42.86
VII	13	10	76.92	71.43	74.07
VIII	7	1	14.29	12.50	13.33
IX	2	2	100.00	100.00	100.00
Total	184	149	80.98	—	—

Note: When excluding the rare types VI and VIII (which accounted for only 1.63% and 0.22% of the overall preoperative cohort, respectively), the model’s classification accuracy on the remaining common types in the validation set was 84.8%.

**Table 4:** Preoperative and postoperative CPAK type distribution.

CPAK Type	Preoperative *n*	Preoperative %	Postoperative *n*	Postoperative %
I	535	58.22	33	3.59
II	139	15.13	45	4.90
III	75	8.16	22	2.39
IV	97	10.55	263	28.62
V	38	4.13	283	30.79
VI	15	1.63	52	5.66
VII	9	0.98	137	14.91
VIII	2	0.22	75	8.16
IX	9	0.98	9	0.98
Total	919	100.00	919	100.00

**Table 5:** Preoperative-to-postoperative CPAK type transition matrix (row percentages, %).

Preop\Postop	I (%)	II (%)	III (%)	IV (%)	V (%)	VI (%)	VII (%)	VIII (%)	IX (%)
I	4.86	5.05	0.75	32.90	27.48	3.36	17.01	7.66	0.93
II	0.72	5.76	5.04	26.62	43.88	7.91	4.32	5.04	0.72
III	6.67	9.33	6.67	8.00	36.00	18.67	1.33	12.00	1.33
IV	1.03	0.00	3.09	29.90	20.62	4.12	30.93	10.31	0.00
V	0.00	0.00	5.26	28.95	34.21	5.26	13.16	7.89	5.26
VI	0.00	0.00	6.67	0.00	46.67	20.00	6.67	20.00	0.00
VII	0.00	11.11	0.00	11.11	44.44	0.00	22.22	11.11	0.00
VIII	0.00	50.00	0.00	50.00	0.00	0.00	0.00	0.00	0.00
IX	0.00	11.11	0.00	22.22	44.44	0.00	11.11	11.11	0.00

**Table 6:** Covariate balance before and after inverse probability weighting.

Covariate	Before Weighting (SMD)	After Weighting (SMD)
Age (years)	0.18	0.04
Sex (male)	0.22	0.05
BMI (kg/m^2^)	0.14	0.03
Preoperative K-L grade (grade 4)	0.19	0.06
Surgical technique (robotic)	0.20	0.07

**Table 7:** Comparison of clinical outcomes after inverse probability weighting.

Outcome	Stable (Weighted *n* ≈ 84.5)	Alignment-Changed (Weighted *n* ≈ 110.8)	Joint Line-Changed (Weighted *n* ≈ 373.5)	Mixed-Changed (Weighted *n* ≈ 332.2)	*p*-Value
Satisfaction, *n* (%)	77.8 (92.11%)	102.1 (92.11%)	339.6 (90.93%)	309.9 (93.29%)	0.93
KSS	151 (143–156)	151 (144–156)	152 (147–158)	152 (146–156)	0.64
WOMAC score	20 (11–27)	18 (13–24)	16 (12–22)	18 (11–25)	0.17
FJS	61.8 ± 19.3	68.4 ± 18.8	63.3 ± 21.5	66.8 ± 18.4	0.14

Note: Effective sample sizes after inverse probability weighting are shown. Weighted Kruskal–Wallis tests and weighted chi-square tests were used for comparisons.

## Data Availability

The data presented in this study are available on request from the corresponding author due to privacy.

## Supplementary Materials

The following supporting information can be downloaded at: https://www.mdpi.com/article/10.3390/diagnostics16091389/s1, Table S1. Preoperative and postoperative CPAK type distribution in the development set (*n* = 827); Table S2. Preoperative-to-postoperative CPAK type transition matrix in the development set (row percentages, %), *n* = 827; Table S3. Transition pattern grouping in the development set; Table S4. Comparison of clinical outcomes among transition pattern groups in the development set only (*n* = 827), without inverse probability weighting.
