# Supplementary material for: Deep Learning-Based Full-Process Automatic CPAK Classification System and Its Application in the Analysis of Alignment Outcomes Before and After Knee Arthroplasty
Source: Diagnostics (Basel). 2026 May 3;16(9):1389. doi: 10.3390/diagnostics16091389 (PMC13163821; doi:10.3390/diagnostics16091389)
Supplement: Supplementary file 1 [file diagnostics-16-01389-s001.zip › diagnostics-4246761-supplementary-material.pdf]

Supplementary Material

Sensitivity analysis using only the development set (n = 827)

Table S1. Preoperative and postoperative CPAK type distribution in the development set (n = 827).

CPAK Type	Preoperative n	Preoperative %	Postoperative n	Postoperative %
I	481	58.16%	30	3.63%
II	125	15.11%	40	4.84%
III	67	8.10%	20	2.42%
IV	87	10.52%	237	28.66%
V	34	4.11%	255	30.83%
VI	13	1.57%	47	5.68%
VII	8	0.97%	123	14.87%
VIII	2	0.24%	67	8.10%
IX	8	0.97%	8	0.97%
Total	827	100%	827	100%

Table S2. Preoperative-to-postoperative CPAK type transition matrix in the development set (row percentages, %). n=827

Preop\Postop	I(%)	II (%)	III(%)	IV(%)	V (%)	VI(%)	VII(%)	VIII(%)	IX(%)
I (n=481)	4.78	4.99	0.83	32.85	27.44	3.33	17.05	7.69	1.04
II (n=125)	0.80	5.60	4.80	26.40	44.00	8.00	4.00	4.80	1.60
III (n=67)	5.97	8.96	5.97	7.46	35.82	19.40	1.49	11.94	2.99
IV (n=87)	1.15	0.00	3.45	29.89	20.69	4.60	31.03	9.20	0.00
V (n=34)	0.00	0.00	5.88	29.41	35.29	5.88	11.76	8.82	2.94
VI (n=13)	0.00	0.00	7.69	0.00	46.15	23.08	7.69	15.38	0.00
VII (n=8)	0.00	12.50	0.00	12.50	37.50	0.00	25.00	12.50	0.00
VIII (n=2)	0.00	50.00	0.00	50.00	0.00	0.00	0.00	0.00	0.00
IX (n=8)	0.00	12.50	0.00	25.00	37.50	0.00	12.50	12.50	0.00

Table S3. Transition pattern grouping in the development set.

Group	n (%)
Stable (preoperative and postoperative types identical)	68 (8.22%)
Alignment-changed (only aHKA classification changed)	103 (12.45%)
Joint line-changed (only JLO classification changed)	347 (41.96%)
Mixed-changed (both aHKA and JLO classifications changed)	309 (37.37%)
Total	827 (100%)

Table S4. Comparison of clinical outcomes among transition pattern groups in the development set only (n=827), without inverse probability weighting.

Outcome	Stable (n=68)	Alignment-changed (n=103)	Joint line-changed (n=347)	Mixed-changed (n=309)	P-value
Satisfaction, n (%) satisfied	62 (91.18%)	96 (93.20%)	317 (91.35%)	289 (93.53%)	0.72>0.05
KSS score, median (IQR)	152 (144–157)	151 (143–156)	152 (147–158)	152 (145–156)	0.71>0.05
WOMAC score, median (IQR)	19 (12–26)	18 (12–24)	16 (11–22)	18 (10–24)	0.11>0.05
FJS score, mean ± SD	62.5 ± 19.1	67.9 ± 18.6	63.8 ± 21.2	66.2 ± 18.5	0.13>0.05

Note: These results are based on the development set (n=827) and represent unweighted raw data, which explains the slight numerical differences compared to Table 7 (full cohort, weighted).
